# The Mirror of Mind: Visualizing Mental Representations of Self Through Reverse Correlation

**DOI:** 10.3389/fpsyg.2020.01149

**Published:** 2020-06-12

**Authors:** Kibum Moon, SoJeong Kim, Jinwon Kim, Hackjin Kim, Young-gun Ko

**Affiliations:** Department of Psychology, Korea University, Seoul, South Korea

**Keywords:** self-image, self-perception, facial images, visual representations, classification images, self-esteem, social anxiety, extraversion

## Abstract

The reverse correlation (RC) method has been widely used, because it allows visualization of mental representations without *a priori* assumptions about relevant dimensions. We employed the RC method to visualize mental representations of self and examined their relationships with traits related to self-image. For this purpose, 110 participants (70 women) performed a two-image forced choice RC task to generate a classification image of self (self-CI). Participants perceived their self-CIs as bearing a stronger resemblance to themselves than did CIs of others (filler-CIs). Valence ratings of participants who performed the RC task (RC sample) and of 30 independent raters both showed positive correlations with self-esteem, explicit self-evaluation, and extraversion. Moreover, valence ratings of independent raters were negatively correlated with social anxiety symptoms. On the other hand, valence ratings of the RC sample and independent raters were not correlated with depression symptoms, trait anxiety, or social desirability. The results imply that mental representations of self can be properly visualized by using the RC method.

## Introduction

Self-image is defined as a subjective perception of oneself, affecting one’s thoughts, feelings, and behavior to a great extent (Coon, 1997). Self-image is commonly known to be in a verbal form. For example, a person with a positive self-image associates words like “Nice,” “Competent,” or “Attractive” with oneself (Amos et al., 1997). It can also be in a visual form, when people conceive images of themselves. Among those images, a facial self-image is claimed to be a fundamental factor of self-identity, self-recognition, and self-awareness, by which one distinguishes oneself from others (Keenan et al., 2000; Lou et al., 2004). However, very little is known about the visual aspect of facial self-image. To understand self-image more thoroughly, we aimed to visualize mental representations of self by using a technique called *reverse correlation* (RC) and examine their relationships with features relevant to self-image.

The RC method is a data-driven approach to creating visual proxies of mental representations (Mangini and Biederman, 2004; Dotsch and Todorov, 2012; Brinkman et al., 2017). In a typical RC image classification task, a large set of facial stimuli is presented to participants in pairs. These facial stimuli are produced by superimposing random noise patterns on a single base facial image. In each trial, participants select one from a pair of faces that better resembles the target category. By averaging the selected stimuli across many trials, one classification image (CI) is generated (for technical details, see Brinkman et al. (2017), who suggested interpreting a CI as a visual form of the internal representations of interest).

In the field of psychology, the RC method has been widely used to visually identify diagnostic features that are involved in social judgments (Dotsch and Todorov, 2012; Brinkman et al., 2017). For example, it has been used to classify features diagnostic for race: Chinese faces (Dotsch et al., 2008), Moroccan faces (Dotsch et al., 2008, 2011), and Black and White faces (Krosch and Amodio, 2014). It has also been employed to classify features diagnostic for personality traits. Dotsch and Todorov (2012) found that diagnostic facial features that are key to the judgment of trustworthiness were a subtle smile and femininity, and dominance was also related to facial masculinity. In another line of research, the RC method has been employed to examine the associations between the distortion in mental representation and pre-existing knowledge or prejudice. For instance, Dotsch et al. (2008) found that the higher the Dutch participants’ level of implicit prejudice toward Moroccans, independent raters rated the CIs of typical Moroccan faces as more criminal and less trustworthy. Imhoff et al. (2013) also assessed the participant’s mental representation of two occupation groups: male nursery teachers and managers. The CIs of nursery teachers were evaluated as warmer but less competent than those of managers.

An RC task has an important strength of incorporating participants’ spontaneous use of information, because they can freely adopt criteria that are important for their judgments about the stimuli (Brinkman et al., 2017). For example, when asked to select faces that bore a stronger resemblance to themselves, some may place greater weight on the eyes or noses of the presented face, but others may focus on somewhat ambiguous factors, such as emotional impressions or trustworthiness in faces. Some participants may not even be aware of the features they adopt to make such judgments. Because the RC method allows participants to make spontaneous and instinctive decisions, *a priori* assumptions about related dimensions are not needed to visualize a participant’s self-image. In this vain, Brinkman et al. (2017) suggested that the RC method could be used to visualize how individuals implicitly see themselves.

How people conceive themselves is an important psychological feature, because it can be closely related to personality traits and the patterns of interpersonal relationships. For instance, Epley and Whitchurch (2008) showed that participants who had a more positive sense of self-worth were more likely to recognize an attractively enhanced version of their face as their own than others. Meanwhile, Buhlmann et al. (2008) reported that people with body dysmorphic disorder (BDD) perceived themselves as less attractive compared to how independent raters perceived them. Similarly, patients with social phobia possessed negatively biased self-image rather than a realistic portrayal of how they come across (Clark and Wells, 1995). Moreover, Hirsch et al. (2006) reported that people who were asked to rehearse negative self-image perceived their performance in social situations more poorly than those who were asked to rehearse positive self-image.

Application of the RC method in the dimension of self-image can give additional knowledge by making the ineffable explicit as a form of image which cannot be easily captured by semantic categories (Mangini and Biederman, 2004). The RC task can generate a visual proxy for self-image, whereas traditional assessments of self-image have been focused on verbalized evaluation, such as self-report measures. For example, Amos et al. (1997) measured self-image by directly asking participants to rate themselves on 19 traits using a Likert scale. Though verbal assessments can reveal important aspects of self-image, they would miss out on a visual aspect. This can also be true for indirect measures. For example, Implicit measures assess the association between semantic categories and target (e.g., the Implicit Association Test; Greenwald et al., 1998; Greenwald and Farnham, 2000). These measures can inform automatic attitudes toward self but cannot *show* how one’s self-image looks like.

Although the RC method has yielded notable findings in the area of social perception, an RC task has not yet been actively used to visualize mental representation of self. In a pioneering study, Imhoff and Dotsch (2013) generated CIs of self, a national in-group (German), and a superordinate group (European). They found that self-image and images of their in-group were independently projected into the visual representation of the superordinate group. However, they examined neither the individual differences in the CIs of self nor its associations with traits relevant to self-image. In fact, to our knowledge, the RC method has not yet been used to examine self-image.

In this study, we aim to visualize mental representations of self by using a reverse-correlation task and examining their relationships with traits related to self-image. Our hypotheses are as follows. First, people would perceive their CIs as bearing a stronger resemblance to themselves than would CIs of others. Second, CI valence rated by self and independent raters would be positively correlated with self-esteem, explicit self-evaluation, and extraversion, but negatively with social anxiety symptoms. Self-esteem is a long-established variable associated with positive self-image (Rosenberg, 1965). According to Oikawa et al. (2012), evaluation of one’s own face and self-esteem are linked at the neural level. Also, previous findings support that socially anxious individuals tend to have negative self-images, as it is known to be a maintaining factor of social anxiety disorder (see for review Ng et al., 2014). We postulated that extraversion (X) of the HEXACO model would be positively correlated with valence ratings of self-CIs, because multiple studies have shown stable associations between extraversion, self-esteem, and the quality of interpersonal relationships (Visser and Pozzebon, 2013; Aghababaei et al., 2016). In addition to the main hypotheses, we explored the relationships between valence ratings of self-CIs and psychological indices, including depression symptoms, trait anxiety, and social desirability.

## Methods

### Participants and Design

We recruited two separate samples: one that performed the RC task (RC sample) and a sample of independent raters. The RC sample included 110 students (70 women). Each participant in the RC sample produced one self-CI. The mean age of this sample was 22.90 (*SD*_*age*_ = 3.09; age-range: 18–34). The RC sample received a gift card equivalent to $13 for their participation. Additionally, 60 participants were recruited as independent raters (30 women; *M*_*age*_ = 25.17, *SD*_*age*_ = 3.75; age-range: 19–35) to acquire objective evaluations of the valence of the CIs created by the RC sample. They received $10 for their participation.

We used deception in introducing the aim of the study to blind the participants to the hypotheses and then debriefed them after the experiment. We introduced that the aim of this study is to examine the way of inferring personality traits from artificially generated facial images. This study was approved by the Institutional Review Board (IRB).

### Materials and Procedures

#### Image-Creation Phase

**FIGURE 1:**
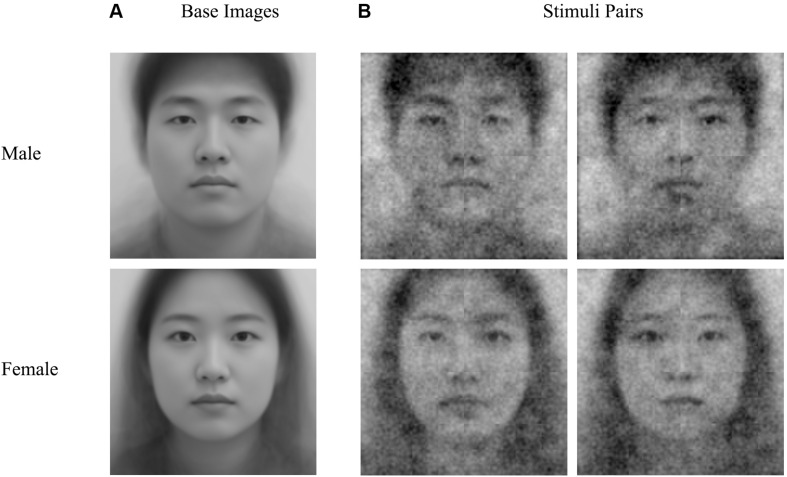
**(A)** Base images and **(B)** examples of stimuli pairs used in the reverse correlation task.

After completing self-report questionnaires, the RC sample performed a two-image forced choice RC task (Dotsch and Todorov, 2012) to generate a classification image of the self. Presented images were a one-base facial image (a morphed composite of 100 faces for each sex) with superimposed random grayscale visual noise (see Figure 1). Every grayscale visual noise was generated by averaging five layers of sinusoid patches. Each patch consisted of different numbers of sinusoid patterns (12, 48, 192, 768, and 3272; see Dotsch and Todorov, 2012 for details).

**FIGURE 2:**
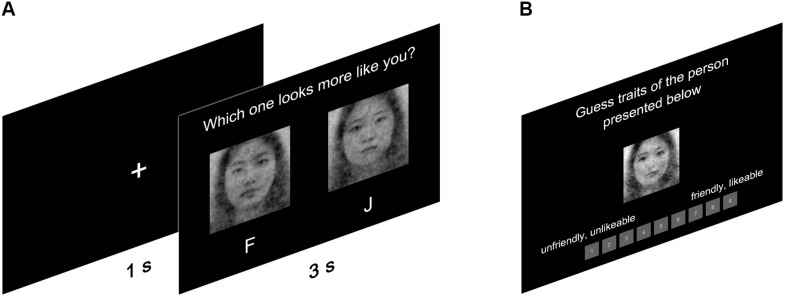
The illustrations of **(A)** image-creation phase and **(B)** image-rating phase.

In 300 trials, participants selected one from two facial stimuli that looked more like themselves (see Figure 2A). Female participants only viewed female faces; males only male faces. Stimuli were presented in random order to the participants, who were forced to make a choice within 3 s. The self-CI of a participant was seamlessly generated by the rcicr package (Dotsch, 2016) in R (R Core Team, 2019) upon the completion of the RC task. The rcicr package averages the noise of the selected images and superimposes the averaged noise on the base image to generate a self-CI for each participant (R codes for generating self-CIs can be found in https://github.com/a072826/The_Mirror_of_Mind). To make sure that participants were unaware of the fact that self-CIs were derived from the image selection task, we exclusively used one computer for performing the RC task and the other for generating the self-CIs. Two computers were synchronized with Internet connection so that self-CIs could be created remotely to minimize the risk of being noticed by the participants. The entire procedure of participation was computerized using psychoPy (Peirce et al., 2019).

#### Image-Rating Phase

Two separate samples rated the CIs generated: the RC sample and the independent rater sample (see Figure 2B). The RC sample rated the CIs that they generated on the valence and on how much it resembled oneself. Also, we presented five filler-CIs with the CI that the participant generated; these filler-CIs for each sex were selected from a pilot study. In the pilot study, 10 men and 10 women (*M*_*age*_ = 25.05, *SD*_*age*_ = 3.14; age-range: 20–29) evaluated trustworthiness and dominance with a 9-point Likert scale from 10 CI stimuli. We selected a set of five CI stimuli per sex considering the average of trustworthiness and dominance of each stimulus set to be similar to the total average. The presentation order of six CIs (five filler-CIs and one self-CI) was randomized to reduce experimental biases. We did not inform participants that they were viewing the CI that they had generated.

Resemblance was rated on 9 Likert points ranging from 1 = *weaker resemblance to myself* to 9 = *stronger resemblance to myself*. Valence was measured with seven items adapted from 14 self-presentational domains (Leary and Allen, 2011)^1^. They were presented on 9-point bipolar scales. Seven items were (a) unfriendly, unlikable vs. friendly, likable; (b) incompetent, unintelligent vs. competent, intelligent; (c) irresponsible, undependable vs. responsible, dependable; (d) immoral, unethical vs. moral, ethical; (e) serious, not playful vs. humorous, playful; (f) unattractive, ugly vs. attractive, good-looking; (g) illogical, irrational vs. logical, reasonable. All items were presented in random order. The seven valence items were averaged to calculate one valence rating. All eight items (self-resemblance and valence) were presented in random order. Participants needed to answer all eight items for each CI presented in the middle of the monitor before moving to the next CI.

The independent raters who were blinded to the study hypotheses also rated the valence of the CIs generated by the RC sample with the seven items listed above. We randomly assigned the independent raters into two groups taking sex ratio into account. Each group evaluated 55 images out of 110 self-CIs. The presentation orders of the CI and of the valence items per CI were randomized across the independent raters. After answering all valence items, independent raters evaluated the next CI.

#### Self-Image Relevant Variables

##### Rosenberg self-esteem scale (RSE)

We used the RSE (Rosenberg, 1965) to measure self-reported global self-esteem. The RSE consists of 10 5-point Likert-scale items (1 = *not very true of me* to 5 = *very true of me*). We used the Korean version of RSE (Lee and Won, 1995).

##### Social interaction phobia scale (SIPS)

The SIPS was employed to assess social anxiety symptoms (Carleton et al., 2009). The scale consists of 14 5-point Likert-scale items (0 = *Not at all characteristic of me* to 4 = *Extremely characteristic of me*). Higher scores indicate a higher degree of social anxiety symptoms. We used the Korean version of the SIPS (Kim et al., 2013).

##### Explicit self-evaluation

To assess explicit self-evaluation, we asked the RC sample to rate how they evaluate themselves using seven items from 14 self-presentational domains (Leary and Allen, 2011). These seven items were also used for the valence ratings of CIs.

##### Extraversion

To assess extraversion, we used 10 items from the HEXACO-60 (Ashton and Lee, 2009)^2^. The HEXACO-60 is scored on a 5-point Likert scale (*1* = *strongly disagree* to *5* = *strongly agree*). The extraversion dimension of the HEXACO-60 consists of four subscales: social self-esteem, social boldness, sociability, and liveliness. We used the Korean version of the HEXACO-60 (Lee and Ashton, 2013).

#### Control Variables

##### Center for epidemiological studies depression scale (CES-D)

The CES-D is a 20-item measure to assess depressive symptomatology (Randloff, 1977). The inventory is scored on a 4-point Likert-scale ranging from 0 = rarely or none of the time (less than 1 day) to 3 = most or all of the time (5–7 days) to assess how often participants felt depressed during the past week. Higher scores indicate more depressive symptoms. We used the Korean version of CES-D (Chon et al., 2001).

##### Taylor manifest anxiety scale (TMAS)

Taylor (1953) developed the Manifest Anxiety Scale (MAS) to measure chronic anxiety symptoms, and Bendig (1956) developed the shortened version of the original scale. The TMAS consists of 20 binary items. We used the Korean version of the TMAS (Lee, 2000).

##### Marlowe-crowne social desirability scale (MCSDS)

The MCSDS is a self-report scale developed by Crowne and Marlowe (1960) to measure the tendency to appear socially desirable. The MCSDS consists of 33 binary items. We used the Korean version of MCSDS (Lee, 2000).

## Results

### Resemblance Rating

For raw data, please see Supplementary Material S1. To test whether participants perceived their own CI as bearing a stronger resemblance to themselves than did filler CIs, we performed a one-way (target: self-CI vs. filler-CIs) repeated measure analysis of variance (RM ANOVA) with resemblance rating as a dependent variable. RM ANOVA was performed due to differing numbers of CIs for each target (one vs. five). There was a significant main effect of target, *F*(1, 109) = 124.38, *p* < 0.001, η*_*p*_*^2^ = 0.53, with a stronger resemblance rating for self-CI (*M* = 5.89, *SD* = 1.93, 95% CI = 5.61, 6.17) than for filler-CIs (*M* = 3.76, *SD* = 2.00, 95% CI = 3.48, 4.04). The resemblance rating was significantly correlated with valence ratings of the RC sample on their own self-CIs, *r* = 0.33, *p* < 0.001. Specifically, resemblance rating was significantly correlated with competency (*r* = 0.46, *p* < 0.001), reliability (*r* = 0.41, *p* < 0.001), and reasonableness (*r* = 0.33, *p* < 0.001) among the seven valence items. Meanwhile, the resemblance rating was significantly correlated neither with the valence ratings of self-CIs evaluated by independent raters (*r* = −0.02, *p* = 0.598) nor with the other study variables (|*r*| *s* = 0.01 ∼0.13, *p* = ns), except for the valence ratings evaluated by the RC sample.

### Valence Rating

**FIGURE 3:**
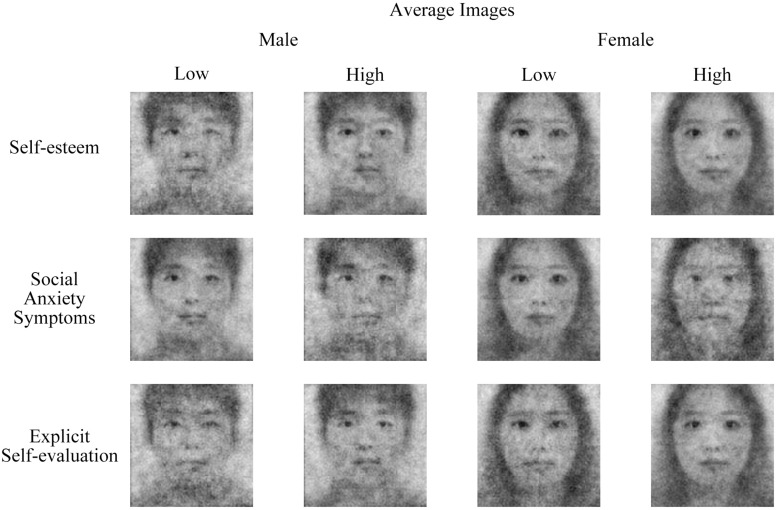
The average self-CIs (classification images of self) by low (−1 SD) and high (+1 SD) groups of self-esteem, social anxiety symptoms, and explicit self-evaluation.

The basic statistics and correlations are shown in Table 1. Reliabilities were acceptable for all measures, with the lowest being 0.72 for MCSDS (Marlowe-Crowne Social Desirability Scale). The valence ratings of self-CIs evaluated by the RC sample and independent raters were positively correlated. For the descriptive purpose, we averaged the resulting self-CIs by high and low groups of self-esteem, social anxiety symptoms, and explicit self-evaluation (see Figure 3). To test the main hypothesis, we investigated the association between the valence ratings of CIs and variables related to self-image. As expected, the valence ratings of the RC sample and independent raters were positively associated with self-esteem, explicit self-evaluation, and extraversion. In addition, the valence ratings of the independent raters were negatively correlated with social anxiety symptoms. Meanwhile, the valence ratings of neither the RC sample nor the independent raters were correlated with depression symptoms, trait anxiety, and social desirability. On the other hand, all of the self-reported measures included in the analysis were significantly correlated to each other, |*r*| *s* = 0.28 ∼0.76, *p* < 0.01.

**TABLE 1:** Means, standard deviations, Cronbach’s αs, and correlations among study variables.

	1	2	3	4	5	6	7	8	9
1. VR_*RC*_	−								
2. VR_*IR*_	0.59***	−							
3. RSE	0.23*	0.24*	−						
4. SIPS	–0.14	−0.27**	−0.44***	−					
5. ES	0.28**	0.44***	0.58***	−0.51***	−				
6. X	0.25**	0.37***	0.71***	−0.61***	0.61***	−			
7. CESD	–0.11	–0.03	−0.73***	0.43***	−0.41***	−0.51***	−		
8. TMAS	–0.14	–0.18	−0.76***	0.59***	−0.52***	−0.72***	0.71***	−	
9. MCSDS	0.04	0.11	0.38***	−0.25**	0.36***	0.28**	−0.29**	−0.41***	−
*M*	5.58	4.88	29.47	12.54	6.36	30.76	16.98	8.18	15.82
*SD*	1.09	0.73	5.88	7.46	0.89	6.89	9.77	5.37	4.8
Cronbach’s α	0.78	0.92	0.89	0.84	0.73	0.85	0.91	0.89	0.72

N = 110. VR_*RC*_, Valence Ratings of the Reverse Correlation sample; VR_*IR*_, Valence Ratings of the Independent Raters; RSE, Rosenberg Self-Esteem Scale; SIPS, Social Interaction Phobia Scale; ES, explicit self-evaluation; X, the Extraversion dimension of the HEXACO; CES-D, Center for Epidemiological Studies Depression Scale; TMAS, Taylor Manifest Anxiety Scale; MCSDS, Marlowe-Crowne Social Desirability Scale. *p < 0.05, **p < 0.01, ***p < 0.001.

## Discussion

Using the RC image classification task (Dotsch and Todorov, 2012), we visualized the mental representation of self. Our data provide evidence that CIs can be regarded as valid proxies of facial self-images. Participants perceived their self-CIs as bearing a stronger resemblance to themselves than did filler CIs, without knowing that the self-CI was an image that they had created via the RC task. Also, in line with our hypothesis, the valence ratings of self-CIs were significantly associated with variables relevant to self-image, including self-esteem, social anxiety symptoms, explicit self-evaluation, and extraversion.

Consistent with the existing literature, our findings suggest that self-image is associated with self-esteem (Rosenberg, 1965; Oikawa et al., 2012) and social anxiety symptoms (Hirsch et al., 2003, 2004). Our findings extend previous research by illustrating that mental representation of self can be visualized through the RC method. It is shown that individuals’ self-images may differ in terms of valence, and this difference can be reliably evaluated across independent raters. Moreover, the data imply that the valence of a mental representation of self pertains to the attitude toward self and the social interaction patterns that one shows.

We also found that the valence ratings of self-CIs were not associated with self-reported depressive symptoms, trait anxiety, or social desirability. On the other hand, all the self-reported variables in this study were correlated with each other. Paulhus (1984) suggested self-deception and impression management as two main contributing factors of self-report bias. These two factors were associated with social desirability. Given the close correlations between social desirability and self-reported measures, it is interesting that the valence ratings of self-CIs did not show significant correlations with social desirability along with depressive symptoms, and trait anxiety. This may imply an advantage of examining self-CIs over self-reported measures, because they allow the researchers to assess participants’ perception of self with less chances of being biased by certain response patterns or social desirability.

It has been reported that distortion in self-perception is a key factor of psychiatric symptoms such as social anxiety disorder (Clark and Wells, 1995; Hirsch et al., 2003, 2004, 2006) or BDD (Buhlmann et al., 2008). Thus, clinicians assess how patients perceive themselves to evaluate the severity of the symptoms and to examine the effectiveness of the therapeutic interventions. Our research suggests the clinical usefulness of using the RC method as a tool to assess how patients see themselves, which may be hard to depict in words.

There may be a concern that participants’ selection of images might be determined solely by the physical resemblance of the presented stimuli. However, we believe that the participants would have considered aspects other than facial resemblance such as emotional impression and trustworthiness in selecting images that looked more like themselves, based on the results of this study and prior research. The valence ratings of self-CIs evaluated by independent raters were not significantly correlated with the resemblance rating, but the valence ratings by independent raters showed significant associations with features related to self-image. On the contrary, the resemblance rating was not correlated with any of the variables related to self-image. The fact that valence ratings by independent raters provided the independent and incremental information about self-image over resemblance rating suggests that participants were less likely to rely exclusively on physical resemblance while performing the RC task. Comparably, previous studies have demonstrated that resemblance judgment was associated with the attitude toward target apart from facial resemblance. For example, women who kept passionate relationships tended to idealize their romantic partners’ facial appearance as more attractive and trustworthy than women who kept less passionate relationships (Gunaydin and DeLong, 2015). Similarly, the more positive automatic attitudes people have toward themselves, the more they select attractively modified images as their actual image (Epley and Whitchurch, 2008).

### Limitations

The limitations of this study are as follows. First, because we employed only self-reported measures to measure the validity of self-CIs, variables that were significantly associated with valence ratings of self-CIs were also closely related to self-reported measures. Thus, the incremental validity of self-CI can be further explored in future studies. The result of Epley and Whitchurch (2008) suggests that visual representations of self may be more closely related to implicit measures than to explicit measures. Therefore, future research can include both implicit and self-reported measures to examine the incremental validity of CI ratings. Also, since we did not incorporate the physical appearance of participants into our analyses, our data cannot explicitly demonstrate an advantage of evaluating self-CIs over physical appearance. It remains for future studies to take physical appearance into consideration in examining visualized self-representation more thoroughly. Finally, the sample in this study was limited to young adults; therefore, the results may not be applicable to other ages. Future research with a broader age range is needed to generalize the current findings.

## Conclusion

Overall, our results support that mental representations of self can be visualized via the RC method. Participants perceived their self-CIs as bearing a stronger resemblance to themselves than did CIs of others (filler-CIs). The valence ratings of participants (RC sample) and independent raters were associated with variables related to self-image. A remaining issue for future research is about the intrinsic and additional information about self-image that the RC method can provide but that traditional measures and physical appearance cannot.

## Data Availability Statement

The raw data supporting the conclusions of this article is available from the Supplementary Material.

## Ethics Statement

The study was approved by the institutional review board at Korea University (KUIRB-2019-0154-01). The patients/participants provided their written informed consent to participate in this study.

## Author Contributions

KM and YK conceived and designed the experiment. KM and JK performed the experiment. KM, YK, and HK analyzed the data. KM, SK, and JK wrote the manuscript.

## Conflict of Interest

The authors declare that the research was conducted in the absence of any commercial or financial relationships that could be construed as a potential conflict of interest.

## Footnotes

1 We conducted an exploratory factor analysis to reduce the dimensions of the 14 self-presentational domains based on the ratings of independent raters. Seven items with high factor loadings on the valence (trustworthiness) factor were selected and averaged to calculate valence ratings (factor loadings = 0.94 ∼0.88).

2 Participants also answered the other 50 items of the HEXACO-60. However, the other five personality dimensions were not significantly correlated with the valence ratings of self-CIs, |*r*| s < 0.17.

## Supplementary Material

MATERIAL S1 Raw data sets of self-report measurements and of the evaluation of participants’ self-CIs in sheet 1 (total_data) and an example data set of the RC task in sheet 2 (RC_data).
